# Research on optimization of control parameters of gravity shaking table

**DOI:** 10.1038/s41598-023-28171-5

**Published:** 2023-01-20

**Authors:** Keshun You, Huizhong Liu

**Affiliations:** School of Mechanical and Electrical Engineering, Jiangxi University of Science and Technology, Ganzhou, 341000 China

**Keywords:** Electrical and electronic engineering, Mechanical engineering

## Abstract

When image processing and machine vision technology are used to extract features from the image of the ore belt of the shaking table, so as to realize the analysis of the processing indictors and mapping of control parameters. To realize the adaptive optimization of the multiple control parameters of the shaking table, it is necessary to have thorough access to the parameters of the internal and external properties of the gravity shaker, such as internal control parameters and external ore zone characteristics, as well as the processing indicators. In this study, information on the multi-scale characteristics of the zone is obtained through a visual experimental system, and the data-driven model of the separation process is constructed to characterize the relationship between the properties of the internal and external parameters of the shaking table, eventually, an adaptive optimization method of control parameters of the shaking table based on maximizing beneficiation efficiency is proposed. The research results show that the data from the geometric characteristics of the ore belts obtained from practical experiments all satisfy the statistical distribution requirements. In the three optimized support vector regression (SVR) models, the sparrow search algorithm optimized SVR (SSA-SVR) has the best comprehensive performance, which overcomes the limits of data samples under objective conditions and basically meets the existing industrial requirements. With these helps, the proposed optimization method has realized the continuous optimization of multiple control parameters of the shaking table, and the optimization results have a good guarantee.

## Introduction

Traditional operation control of shaking table mainly relies on the operators to observe the ore belt of and then determine the operation state through the appearance characteristic information such as the shape and color of the ore belt. The feed water, slope angle, stroke, stroke rate and other operating parameters are adjusted accordingly. Since each person's experience, technical proficiency and sense of responsibility are different, it is only through the human eye to observe the mining belt of the shaking table to judge whether the operating state is normal, and there is a large error in relying on workers to optimize parameters. Therefore, an objective and accurate method is urgently needed to obtain the characteristic parameters of the ore belt of the shaking table and to truly determine the real-time operating state and processing indicators. And finally replace people to realize the optimization and adjustment of control parameters.

With the development of machine vision technology in recent years, vision technology has been widely used in many fields, and it has also been used in the feature extraction of ore belt images. For example, machine vision is used to extract the position information of the concentrate ore belt to realize the automatic interception of the concentrate^1,2^. In addition, there is no more thorough research on the intelligence of the shaking table, at present, the image processing algorithm of grayscale calculus is used to calculate the boundary points of the ore belt, and the characteristics of the boundary line of the ore belt are obtained by fitting, and finally the width and color characteristics of the ore belt are obtained, so as to realize the complete segmentation of the ore belt image^2^, and the multi-threshold color ore belt image segmentation algorithm optimized by krill^3^ and the multi-threshold color ore belt image segmentation algorithm based on the improved algorithm have been applied to the ore belt image segmentation of the shaking table^4^. With the continuous development of deep learning and machine vision, more and more effective deep convolutional neural network models and machine vision image processing algorithms are applied to the process of mineral identification and sorting^5^, For example, the ResNet network model based on deep learning target detection is constructed, and the position coordinates to be detected are identified^6^, it is proved that the deep learning image algorithm has better accuracy than other intelligently optimized threshold segmentation algorithms in processing the image of the ore belt of shaking table. However, the deep learning target detection algorithm can only extract the shallow image features, which just serves the research and development of a simple automatic mining system. Therefore, the feature extraction algorithm of ore belt image based on deep learning semantic segmentation is applied, and achieves very good results, which can extract the multi-scale features of ore belt image with the largest dimension, which will make important foundational work for the control and optimization of the mineral separation process of gravity shaking table^7^.

And the vast majority of researchers' optimization research on control parameters of beneficiation shaking table is relatively one-sided and scattered. Manser et al.^8^ constructed a logistic regression mathematical model to study the influence of shaking table parameters on the separation condition, and Panda, Lopamudra et al.^9^ constructed a prediction model between gravity concentrator and processing index, equipment performance through the method of case study. These studies are only based on the separation conditions and the individual targeting control parameters that affect the separation performance as optimization goals. And a large number of optimization studies of mineral separating process have proved that neural networks have the perfect ability to learn the relationship between complex variables, such as the relationship between the variables of a complex system of the mineral separation process^10^, compared to other linear regression methods, such as the Plitt model^11^ and Richardson–Zaki model^12^, or other slightly more complex nonlinear regression methods of SVM^13^, random forest statistical regression^14^, and other methods^15^. When they are used to build models between complex variable relationships, although neural networks can well build models between complex variable relationships, in the actual mineral separation process of the gravity separation shaking table, due to the limitation of measurement technology and measurement cost, it is difficult to obtain some control parameter values of the shaker equipment, and there are considerable errors, in addition, the acquisition of the true value of these indicators of the shaking table, such as concentrate grade, recovery rate and beneficiation efficiency, etc. which is obtained by testing the pulp sample, it is very time-consuming, labor-intensive and expensive. Therefore, in the mathematically modelling minerals separation process of shaking table, the problem of limited data samples should be taken into account.

In view of the current problems of image recognition and feature extraction of mineral belts, it is necessary to use more appropriate deep learning image processing algorithms to deeply mine the rich multi-scale features of the ore belt images of the shaking table. Simultaneously, a machine learning model with small samples, high precision and excellent gener-alization performance needs to be used to realistically predict the control parameter values and processing indicators of the shaking table through the image of the ore belt, so as to realize dynamic monitoring. Last but not least, it is necessary to combine the previous research results to propose an adaptive optimization method that takes the processing index as the optimization goal and the control parameters of the shaking table and the characteristic parameters of the ore belt as the optimization result, which is an important and necessary step to realize the intelligent beneficiation of shaking table.

In this study, to realize multi-scale feature extraction of ore belt image of the shaking table, the deep learning algorithm was adopted to design a vision-based system of feature extraction of ore belt image, which was finally applied to industrial test system of separating process of shaking table. To realize the real reflection of the internal and external property parameters of the shaking table operation process through the image of the ore belt in the case of small samples, the SSA-SVR is adopted to construct the machine learning model of taking multi-scale image features of the ore belt as the input, control parameters and beneficiation efficiency as the outputs. To complete the integration of the previous research results, and finally realize the self-adaptive optimization of multiple control parameters of the shaking table, an adaptive control parameter optimization method based on the maximization of beneficiation efficiency is adopted, which can automatically maximize the beneficiation efficiency and minimize labor costs according to the actual requirements of the beneficiation plant and the actual performance of the equipment.

## Geometric feature data of ore belts from the experimental system

The ore belt of shaking table has the functions of characterizing the shaking table control parameters and analyzing the processing state. Therefore, the study of the feature extraction of the ore belt in this paper is of great significance to realize the real-time monitoring of the beneficiation index and the optimization of the control parameters. In this study, a deep learning semantic segmentation algorithm based on DeepLab V3 + is used to extra the multi-scale feature of ore belt image. Different from other image edge detection operators and threshold segmentation algorithms, the deep learning image processing algorithm has better recognition and detection performance, which is consistent with the deep learning target detection network model. The difference is that the deep learning semantic segmentation algorithm can accurately obtain the richer image features of the ore belt.

### Experiment introduction

**Figure 1:**
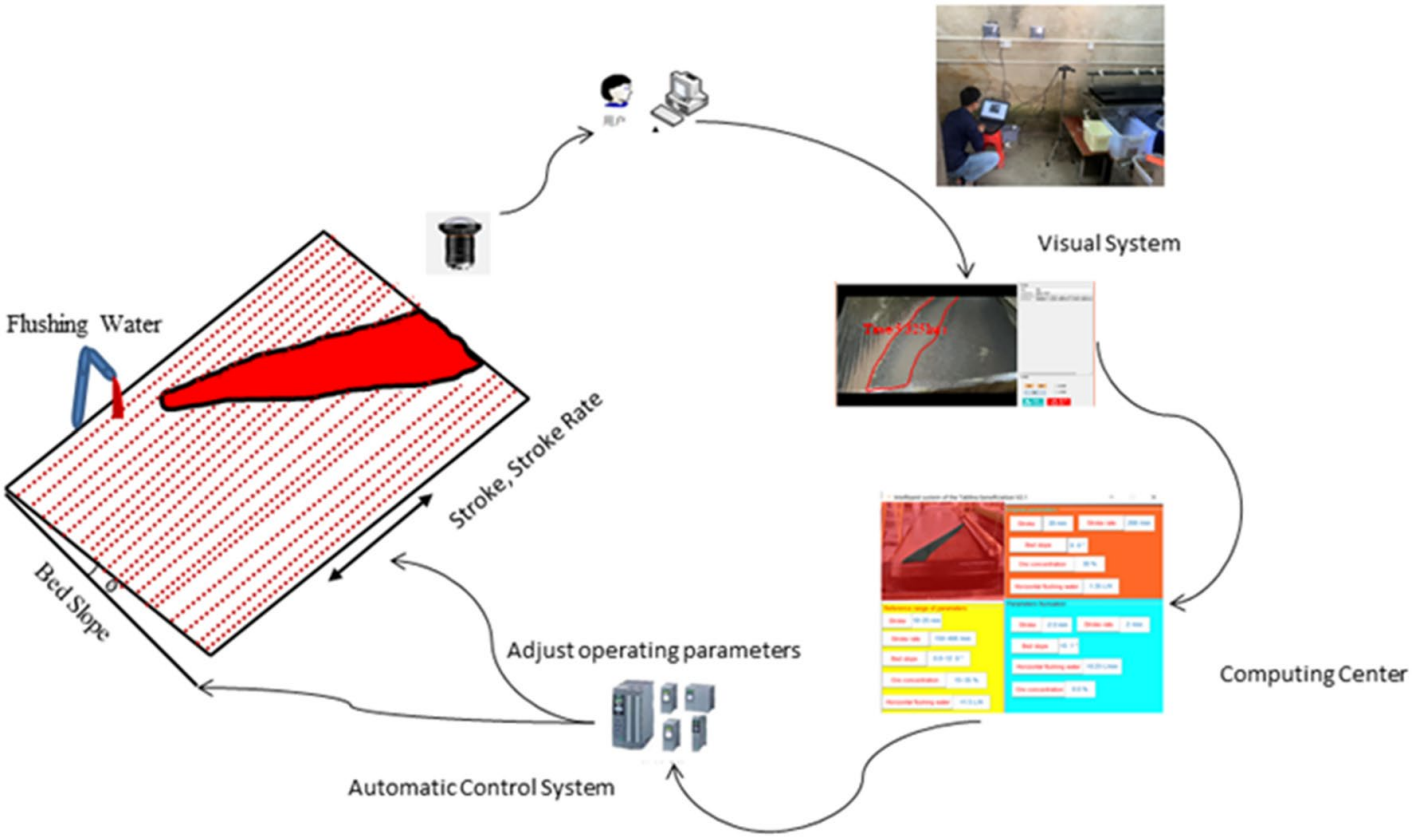
Experiment system for mineral separation and processing of shaking table.

In order to obtain all kinds of data needed to build the relevant machine learning models, we built an experimental system for the mineral processing of shaking table. As shown in Fig. 1, this system is an idealized experimental system. First of all, it is assumed that the ore feeding amount is stable and the ore feeding concentration is constant, and the control parameters such as Feed water, stroke, stroke rate and the lateral slope angle of the bed surface are reasonably adjusted. When the separation process is stable and the stratified area appears on the bed surface, and the water flow is evenly distributed, and the bed surface is free from waves, and the ore is not piled up, and the concentrate is clearly zonal, and the width is thin and the width of the non-mineral area on the bed surface is appropriate, then we can obtain pulp samples that need to be tested. The corresponding concentrates and tailings were obtained as ore samples for analysis of processing indicators.

**Figure 2:**
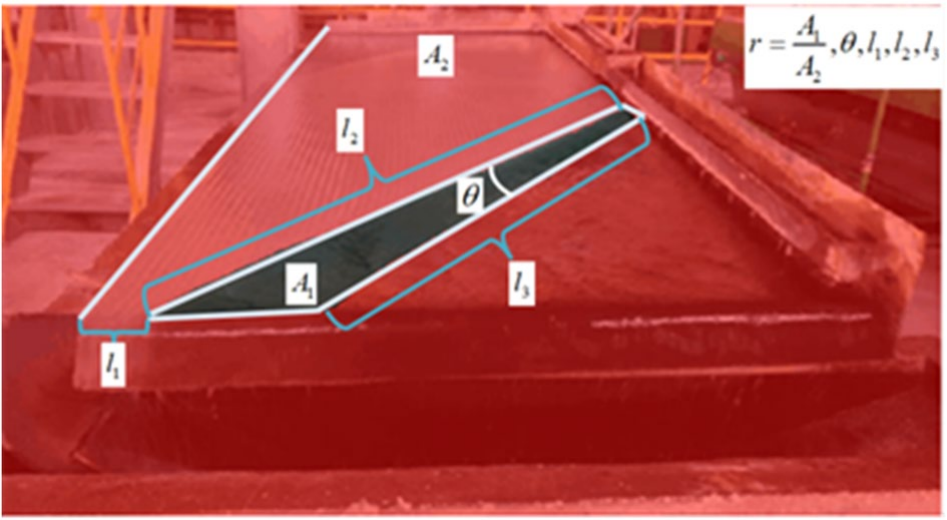
The principle of image feature extraction of multi-scale ore belt.

Before the experiment, to obtain the particle size and grade of the raw ore that meets the experimental conditions, we carried out flotation and screening processes on the raw ore pulp obtained. The purpose of flotation is mainly to remove the interference Molybdenum in the tungsten gravity beneficiation. In the end, we obtained the original feed ore sample with a grade of 2.98% (WO_3_) and a particle size of about 0.3 mm. The useful minerals in the ore sample are tungsten ore and a small amount of tin ore, and the impurities are mainly gangue such as quartz. And the experimental equipment is mainly composed of industrial camera with image data acquisition function, image processing software, wireless communication equipment responsible for data transmission and PLC system for automatic parameter adjustment. The industrial camera monitors the changes of the images of the mining belt in real time, and the designed image processing software draws frames and maps the real-time video. The industrial camera monitors the changes of the ore belt image in real time by the designed image processing software, which draws frames and maps the real-time video. The obtained large amount of image data is imported into the trained deep learning model for prediction, which will be analyzed, extracted and characterized. As shown in Fig. 2, the geometric features of the image are represented as five representative image geometric feature parameters: *r*, $\theta$, *l*_*1*_, *l*_*2*_, *l*_*3*_. In which *A*_*1*_ is the area of the ore belt area, A_2_ is the area of clear water area, the ratio of *A*_*1*_ to *A*_*2*_ is *r*, $\theta$ is the angle between the left and right boundary lines of the ore belt, the length of the left boundary line of the ore belt is *l*_*2*_, the length of the right boundary line of the ore belt is *l*_*3*_, and the distance between the intersection of the left and bottom border lines and the left border line of the shaking table is *l*_*1*_. These five characteristic values characterize the fluctuation of the ore belt from the three dimensions of the point, line and surface of the ore belt, which can closely reflect the influence on the overall operation condition of the beneficiation equipment of shaking table.

### Result analysis of feature extraction of ore belt image based on industrial experiment

In this study, 80 sets of industrial test data are obtained from the experiment system of shaking table as shown in Fig. 1. In the experiment, we reasonably adjust the feed water, slope angle, stroke, stroke rate, and wait for the separation process to be in a stable condition, then obtain the corresponding concentrate and tailings as the pulp samples for analysis of processing indicators, and record the corresponding control parameters and the eigenvalues of ore belt.

**Figure 3:**
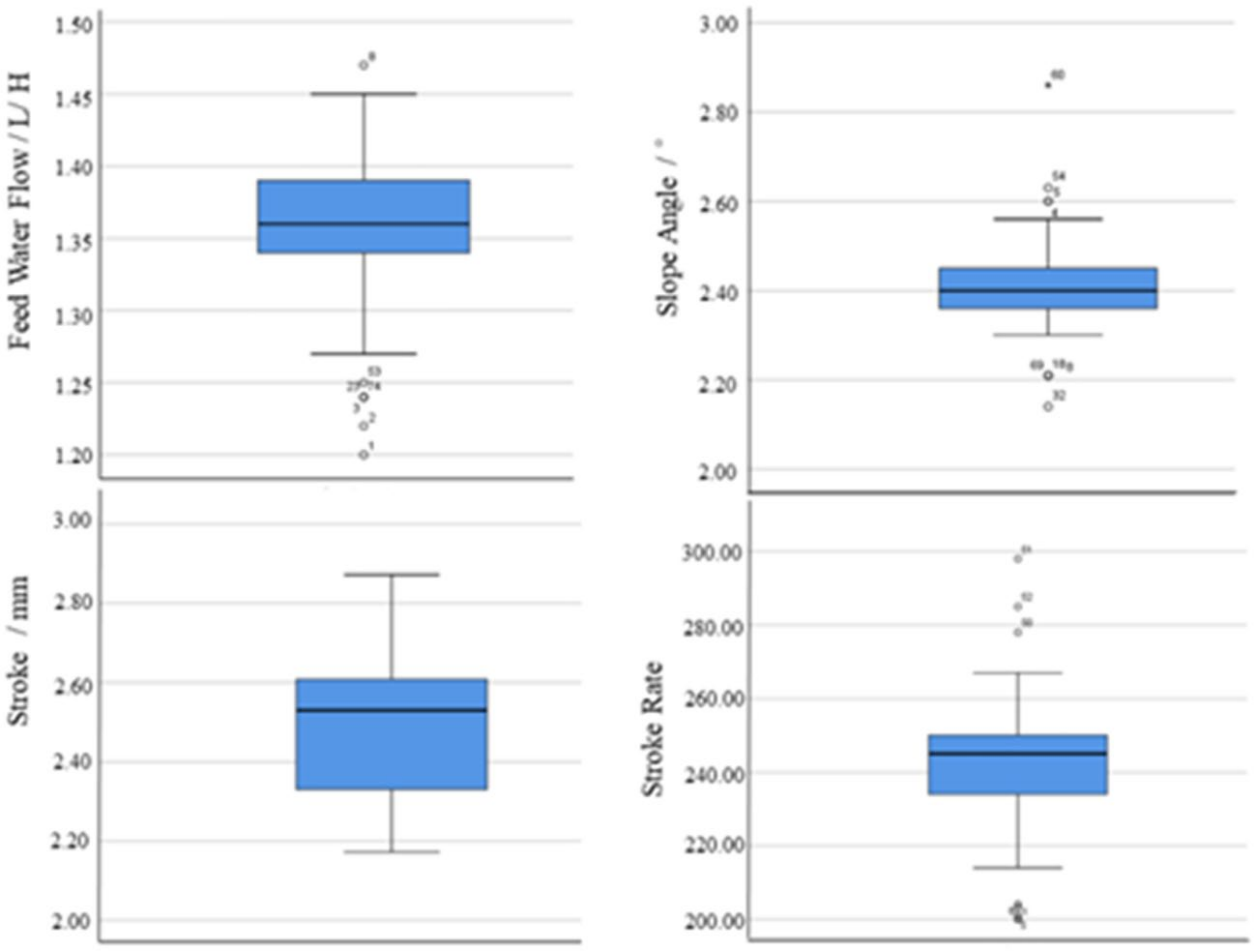
Abnormal analysis of control parameter values.

**Figure 4:**
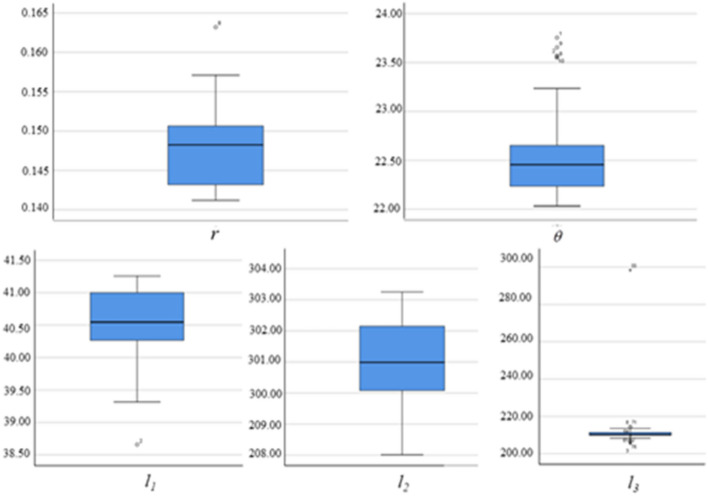
Data analysis of corresponding ore belt characteristic.

As shown in Fig. 3, in the experiment, we made a total of 80 parameter adjustments based on production experience, and we recorded the parameter fluctuations after each parameter adjustment, after each parameter adjustment, we obtained the measured values of the four control parameters in the stable state of the distribution of the ore belt of the shaking table. It is shown in Fig. 4 that we use the above-mentioned vision technology based on deep learning image processing algorithm to extract the eigenvalues of the multi-scale ore belt of the shaking table. In order to analyze whether the adjustment of the control parameters of the shaking table is normal in the industrial experiment, we performed a box diagram analysis based on the statistical principle on the data of 80 groups of control parameter values. Through analysis, it is found that the data distribution of most of the control parameter values fluctuates within a reasonable range, and some control parameters are due to errors in experimental design and measurement, resulting in some deviations in the distribution, which are normal errors in the experiment and will not affect the overall data distribution characteristic. Due to the limited data obtained by ourselves, we retained these 80 sets of data and continued to analyze the extracted eigenvalues of ore belt data set. The analysis found that the data distribution of the ore belt characteristic values was obviously more in line with the box distribution, which indirectly shows that each parameter adjustment is basically carried out when the ore belt is close to normal and stable.

The concentrate slurry and tailings slurry intercepted by the shaking table are respectively dried and sampled, and then the concentrate and tailings obtained in the experiment are subjected to processes such as crushing, shrinking, grinding, etc., and finally a special grade measurement is carried out. This work is greatly constrained by objective conditions such as economy and labor resources.

Concentrate grade and recovery rate of shaking table are conflicting but indispensable evaluation criteria and therefore need to be considered in a comprehensive manner. Given that it is difficult and cumbersome to judge the separation indicators of the shaking table by concentrate grade and recovery rate, it is not conducive to later model construction. So this paper adopts the beneficiation efficiency to characterize the integrated separation state. When there is no separation effect, its value is zero, when the separation effect is ideal, its value is 100%.

1 $$E = \frac{\beta }{\alpha } * \frac{{\left( {\alpha - \theta } \right)\left( {\beta - \alpha } \right)}}{{\left( {\beta - \theta } \right)\left( {\beta_{x} - \alpha } \right)}}$$

2 $$E = \frac{\varepsilon - \gamma }{{1 - {\raise0.7ex\hbox{$\alpha $} \!\mathord{\left/ {\vphantom {\alpha {\beta_{x} }}}\right.\kern-0pt} \!\lower0.7ex\hbox{${\beta_{x} }$}}}}$$

where $\varepsilon$ is the recovery rate and $\gamma$ is the concentrate rate, $\alpha$ is the feed grade, $\beta$ is the concentrate grade, $\theta$ is the tailings grade, $\beta_{x}$ is the percentage of useful components in the pure minerals to be selected, and *E* is the beneficiation efficiency. It can be seen from Eqs. (1, 2) that the beneficiation efficiency can integrate the separation indicators of concentrate grade and recovery rate, and objectively reflect the separation state of the shaking table.

## A data-driven model for characterizing the relationship between internal and external parameters

Due to the limitation of objective conditions, only 80 sets of valid data were obtained in the experimental system. In order to make full use of these sample data, a small sample and high-accuracy data model should be considered when constructing the relevant machine learning model. Although the deep learning convolutional neural network regression algorithm has good learning ability and model accuracy, it requires a large number of data samples for training, which is obviously not suitable for building small-sample machine learning models. Therefore, when building related models, we did not consider particularly complex machine learning algorithms, but chose a more practical support vector regression (SVR) algorithm for data modeling. First of all, considering that support vector machines and support vector regression have been widely used in industrial practice, and the accuracy and stability of their algorithms have become increasingly prominent, they have been well used in the fields of mineral dehydration^16,17^.

### Introduction to the model of the mineral separation process of shaking table

Due to the very complex nonlinear coupling between the mineral zoning and beneficiation efficiency of the shaking table, as well as the control parameters of the shaking table, the suitable relationship models between the mineral zoning and the beneficiation efficiency of the shaking table, as well as the control parameters of the shaking table need to be explored, which requires us to build a machine learning model with excellent learning performance, good generalization performance, and accuracy that can meet industrial requirements.

Support vector machine (SVM) is a learning method based on statistical learning theory and as widely used as deep learning for all kinds of complex tasks^18,19^, which has many advantages such as complete theory, strong adaptability, global optimization, short training time, and good generalization performance. Since the Gaussian kernel function is generally used for training and learning, two important hyperparameters are involved, namely the penalty parameter *c* and the kernel parameter *g*, which have an important impact on the prediction performance of the model. As external parameters to the model, the *c* and *g* parameters are special among the many parameters of an SVM model, as they need to be thought of as set and will not be computed through training. For SVR algorithms that apply SVM for regression tasks, simply using the support vector regression model to learn has certain limitations, mainly because the support vector regression has two important hyperparameters in the training process, *c* and *g*, which respectively determine the accuracy and generalization performance of SVR^20^. Therefore, it is necessary to use an intelligent optimization algorithm to optimize these two hyperparameters, which will greatly improve the learning performance and generalization performance of the model.

However, the Sparrow Search Algorithm (SSA) is an excellent swarm intelligence optimization algorithm^21^, which originates from the behavior of sparrows foraging and avoiding predators. Sparrows are divided into two parts: discoverers and joiners, which are responsible for providing foraging directions and following food sources respectively^22^. Because the sparrow search algorithm has good local search ability, it can overcome the shortcomings of the SVR algorithm in the lack of local optimization ability. Therefore, this paper focuses on the application research of the optimal support vector regression of the sparrow search algorithm (SSA-SVR) in the optimal control of the separating process of shaking table.

In the SSA optimization process, each sparrow position corresponds to one of the solutions, suppose the population formula composed of *n* sparrows is as follows:

3 $$X = \left[ {\begin{array}{*{20}c} {x_{11} } & {x_{12} } & \cdots & {x_{1n} } \\ {x_{21} } & {x_{22} } & \cdots & {x_{2n} } \\ \vdots & \vdots & \vdots & \vdots \\ {x_{n1} } & {x_{n2} } & \cdots & {x_{nn} } \\ \end{array} } \right]$$

where *d* represents the dimension of the problem variable to be optimized. When the sparrows as discoverers are looking for prey, followers will join the team of discoverers, so the number of discoverers and joiners fluctuates in real time and can be converted into each other, but the ratio of the two remains constant. The formula of finder's location update is as follows:

4 $$X_{ij}^{t + 1} = \left\{ \begin{gathered} X_{ij}^{t} + Q \cdot L \,\,\,\,\,\,\,\,\,\,\,\,\,\,\, if \, \,\,\,R_{2} \ge ST \hfill \\ X_{ij}^{t} \cdot \exp \left( { - \frac{i}{{\alpha \cdot iter_{\max } }}} \right) \,\,\,\,\,\,\, if \,\,\,\,\,\,\,\,\,\ others \, \hfill \\ \end{gathered} \right.$$

where *t* represents the current iteration number, *j* = 1, 2, 3,…,*d*, *item*_max_ represents the maximum number of iterations, and $X_{ij}^{t}$ is the current position, $X_{ij}^{t + 1}$ is the updated position, *R*_*2*_ is the warning value, *ST* is the safety value, *Q* is a random number of normally distributed, and *L* is a d-dimensional column vector. When *R*_*2*_ < *ST*, it means that there are no predators around the foraging environment at this time, and the finder can perform a wide range of search operations. If *R*_*2*_ ≥ *ST*, it means that some sparrows in the population have detected a predator and alerted other sparrows in the population, and all sparrows need to fly quickly to other safe places to forage.

The population will randomly select 10% ~ 20% of the sparrows as vigilantes for monitoring and early warning. When the sparrows at the edge of the population perceive danger during the foraging process, they will quickly remind the entire population to act against predation. When the warning value is greater than the safety value, the finder will take the joiner to other safe areas for foraging. The formula of the joiner position update is as follows:

5 $$X_{ij}^{t + 1} = \left\{ \begin{gathered} Q \cdot \exp \left( { - \frac{{X_{w} - X_{ij}^{t} }}{{i^{2} }}} \right) \,\,\,\,\,if \,\,\, others \hfill \\ X_{p}^{t + 1} + \, \left| {X_{ij}^{t} - X_{p}^{t + 1} } \right| \cdot A^{ + } \cdot L \,\,\,\, if \,\,\,\,\, i \le {\raise0.7ex\hbox{$n$} \!\mathord{\left/ {\vphantom {n 2}}\right.\kern-0pt} \!\lower0.7ex\hbox{$2$}} \hfill \\ \end{gathered} \right.$$

where $X_{p}^{t + 1}$ is the optimal position of the finder, *X*_*w*_ is the current global worst value, *A* represents a 1*d matrix, the elements in the matrix are randomly assigned to 1 or -1, and satisfy the formula:

6 $$A^{ + } = A^{T} \left( {AA^{T} } \right)^{ - 1}$$

When *i* > *n/2*, it indicates that the *i*-th joiner with lower fitness value did not get food and is in a very hungry state. At this time, it needs to fly to other places for food to obtain more energy.

As the guide of population foraging, the finder has a higher fitness value and can obtain a wider search area. The joiners follow the discoverers to forage in order to obtain higher fitness values. The fitness value of each sparrow can be expressed as follows:

7 $$F_{X} = \left[ {\begin{array}{*{20}c} {f\left( {\left[ {x_{11} \, x_{12} \, \ldots \, x_{1d} } \right]} \right)} \\ {f\left( {\left[ {x_{21} \, x_{22} \, \ldots \, x_{2d} } \right]} \right)} \\ \vdots \\ {f\left( {\left[ {x_{n1} \, x_{n2} \, \ldots \, x_{nd} } \right]} \right)} \\ \end{array} } \right]$$

At the same time, some joiners may continuously monitor the discoverers and seize food resources to increase their predation rate. When aware of danger, sparrows at the fringes of the flock will quickly move towards a safe area to gain a better position, while sparrows in the middle of the flock will move randomly to get closer to other sparrows. The formula of the early warning sparrow position update is as follows:

8 $$X_{ij}^{t + 1} = \left\{ \begin{gathered} X_{b}^{t} + \beta \cdot \left| {X_{ij}^{t} - X_{b}^{t} } \right| ,\,\,\,\,\,\, if \, f_{i} { > }f_{g} \hfill \\ X_{ij}^{t} + K \cdot \left( {\frac{{\left| {X_{ij}^{t} - X_{w}^{t} } \right|}}{{\left( {f_{i} - f_{w} } \right) + \varepsilon }}} \right) \,\,\,\, if \, f_{i} \, = \, f_{g} \, \hfill \\ \end{gathered} \right.$$

where $X_{b}^{t}$ is the current global optimal value, *β* is used as the step size, which is a random number obeying the normal distribution with mean 0 and variance 1, and *K* ∈ [− 1,1] is also a random number, *f*_*i*_ represents the fitness value of the current sparrow individual, *f*_*g*_ and *f*_*w*_ represent the current global best and the current global worst fitness value respectively, *ε* is a constant, this design is mainly to avoid the denominator from appearing 0. When *f*_*i*_ > *f*_*g*_*,* it means that the sparrow at this time is at the edge of the population and is extremely vulnerable to predators. If *f*_*i*_ = *f*_*g*_, it means that the sparrow in the middle of the population is aware of the danger and needs to get close to other sparrows in order to minimize their risk to be preyed on.

### Data preparation and model building

As an important mineral separation equipment, the shaking table has great potential in optimizing the process and technology of mineral processing^23^, and machine learning will become a powerful method for the control and optimization of the mineral processing^24^. To explore the complex nonlinear relationship mapping between the eigenvalues of ore belt and processing indicators of the beneficiation efficiency, the control parameters of the shaking table, a suitable relationship model between them should be constructed. When constructing the relationship model between the geometric features of ore belt and the control parameters of the shaking table, we use the SSA-SVR algorithm to build the model.

In this paper, 80 sets of data are divided into a 5:3 ratio to divide the training set and the test set, that is, 50 sets of data are used as the training set and 30 sets of data are used as the test set to get a complete machine learning model of separation process of beneficiation shaking table. This is mainly aiming to realize the self-adaptive optimization of the control parameters of shaking table and the intelligent beneficiation shaking table, this plays a huge role in linking the past and the future.

The optimization purpose of the sparrow search algorithm is to find a suitable set of hyperparameters, *c* and *g*, so that the constructed support vector regression model can get the best improvement in the global and local optimization and learning ability. As one of the most important indicators for evaluating machine learning regression models, the mean square error (*MSE*) is the most widely used, and the lower the value, the better performance of the model. Therefore, if *MSE* can be used to evaluate the fitness function of the model, it will fundamentally improve the performance of the model, as shown in Eq. (8).

9 $$MSE = \sqrt {\frac{1}{n}\sum\limits_{i = 1}^{n} {\left| {y_{i} - \overset{\lower0.5em\hbox{$\smash{\scriptscriptstyle\frown}$}}{y}_{i} } \right|}^{2} }$$

where *n* is the number of samples, *i* is the sample number, $$y_{i}$$ is the model predicted value; $$\overset{\lower0.5em\hbox{$\smash{\scriptscriptstyle\frown}$}}{y}_{i}$$ is the true value; $$\left| {y_{i} - \overset{\lower0.5em\hbox{$\smash{\scriptscriptstyle\frown}$}}{y}_{i} } \right|$$ is the absolute value of the error between the predicted value and the true value.

**Figure 5:**
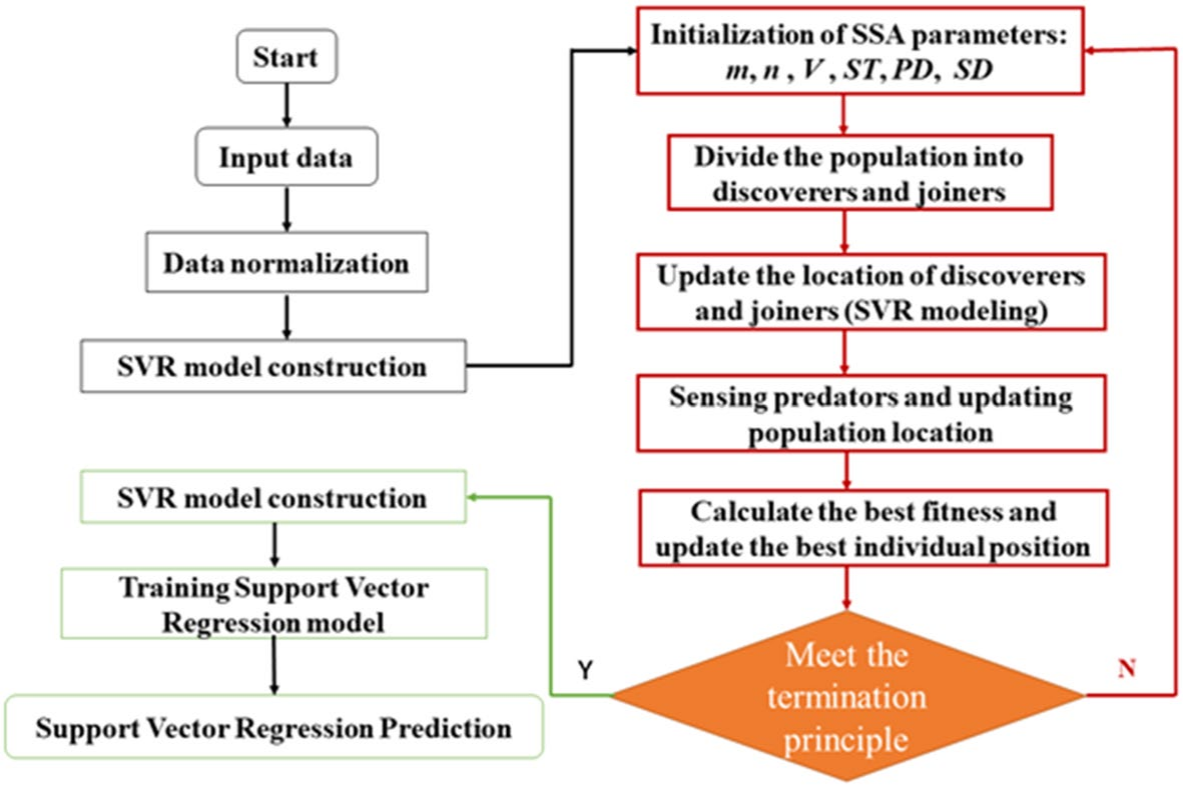
Algorithm flow chart of SSA-SVR.

As shown in Fig. 5, the intelligent swarm algorithm based on SSA performs adaptive optimization for the two hyperparameters of the traditional support vector regression model, which not only fully utilizes the high accuracy and good generalization performance of the SVR model itself, but also makes up for disadvantages of poor global performance. The specific SSA-SVR model construction and operation process is as follows:

1. Read the data, perform data preprocessing, and build an SVR model.
2. The sparrow population parameters are initialized, where *m* is the maximum number of iterations, *n* is the population size, *V* is the cross-validation folded tree, *PD* is the proportion of discoverers, and *SD* is the proportion of sparrows aware of danger. And the value ranges of *c* and *g* are determined. The parameter settings of the sparrow search algorithm are shown in Table 1.
3. Determine the fitness function of the sparrow search algorithm as shown in Eq. (8), and use its value as the amount of food the sparrow searches. According to the principle of the sparrow search algorithm, find the optimal function value, that is, determine the position of the best sparrow individual.
4. The optimal values of parameters *c* and *g* are obtained according to the optimal individual position of the sparrow.
5. After that, the optimal parameters c and g are assigned to SRM for training, and the latest optimized SVR prediction model is obtained.
6. First, the test sample is input, and the prediction model outputs the predicted value of the test sample, and then compares it with the true value and analyzes the error between the two.

**Table 1:** Parameter initialization settings for SSA algorithm optimization.

*m*	*n*	*V*	*ST*	*PD*	*SD*
100	20	3	0.6	0.7	0.2

### Laboratory simulation results

The evaluation of the simulation results of the machine learning regression model mainly lies in analyzing the error between the predicted value and the true value of the model. At present, there are many evaluation indicators for the regression model, and each evaluation indicator contains the specific performance of the model in a certain aspect. In order to analyze the simulation results of the SSA-SVR model for the control parameters and separation conditions of shaking table, the mean square error (*MSE*), mean absolute error (*MAE*), root mean square error (*RMSE*) and mean absolute percentage error (*MAPE*) and the coefficient of determination (*R*^2^) is used to analyze the simulation performance of the model:

10 $$MAE = \frac{1}{n}\sum\limits_{i = 1}^{n} {\left| {y_{i} - \overset{\lower0.5em\hbox{$\smash{\scriptscriptstyle\frown}$}}{y}_{i} } \right|}$$

11 $$RMSE = \sqrt {\frac{1}{n}\sum\limits_{i = 1}^{n} {\left( {y_{i} - \overset{\lower0.5em\hbox{$\smash{\scriptscriptstyle\frown}$}}{y}_{i} } \right)}^{2} }$$

12 $$R^{2} = 1 - \frac{{\sum\nolimits_{i = 1}^{n} {\left( {y_{i} - \overset{\lower0.5em\hbox{$\smash{\scriptscriptstyle\frown}$}}{y}_{i} } \right)^{2} } }}{{\sum\nolimits_{i = 1}^{n} {\left( {y_{i} - \overline{y}_{i} } \right)^{2} } }}$$

13 $$MAPE = \frac{100\% }{n}\sum\limits_{i = 1}^{n} {\left| {\frac{{y_{i} - \hat{y}_{i} }}{{\hat{y}_{i} }}} \right|}$$

where $$\overline{y}_{i}$$ is the true average value, and $$y_{i} - \overset{\lower0.5em\hbox{$\smash{\scriptscriptstyle\frown}$}}{y}_{i}$$ represents the difference between the true value and the predicted value. The smaller the *MSE* and *RMSE*, the better the fitting effect of the model, the higher the *R*^2^, the more realistic the model and the better the fitting effect, the smaller the value of *MAPE*, the better the accuracy of the prediction model, the smaller the *MAE*, the better the simulation error of the model smaller.

**Figure 6:**
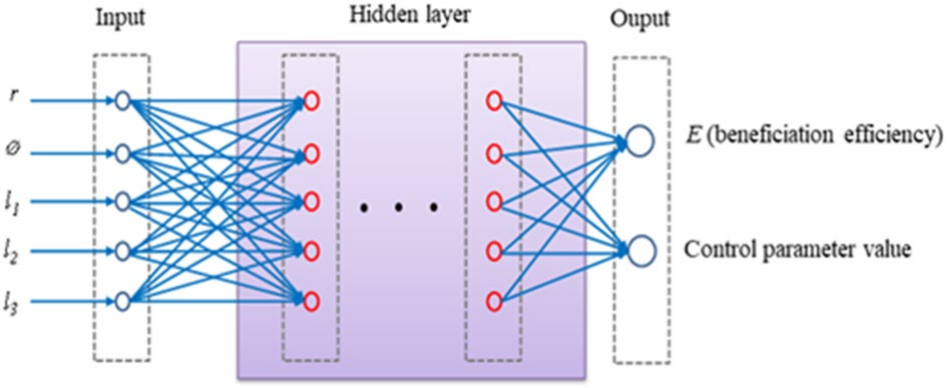
Simulation model of beneficiation shaking table.

As shown in Fig. 6, it shows the principle of the simulation model of the shaking table in the mineral separation process control, which is similar to the machine learning model of the neural network. And it can take the five representative characteristic values of ore belts as input, and take the beneficiation efficiency and control parameters of the shaking table as the output to build the corresponding simulation models.

**Figure 7:**
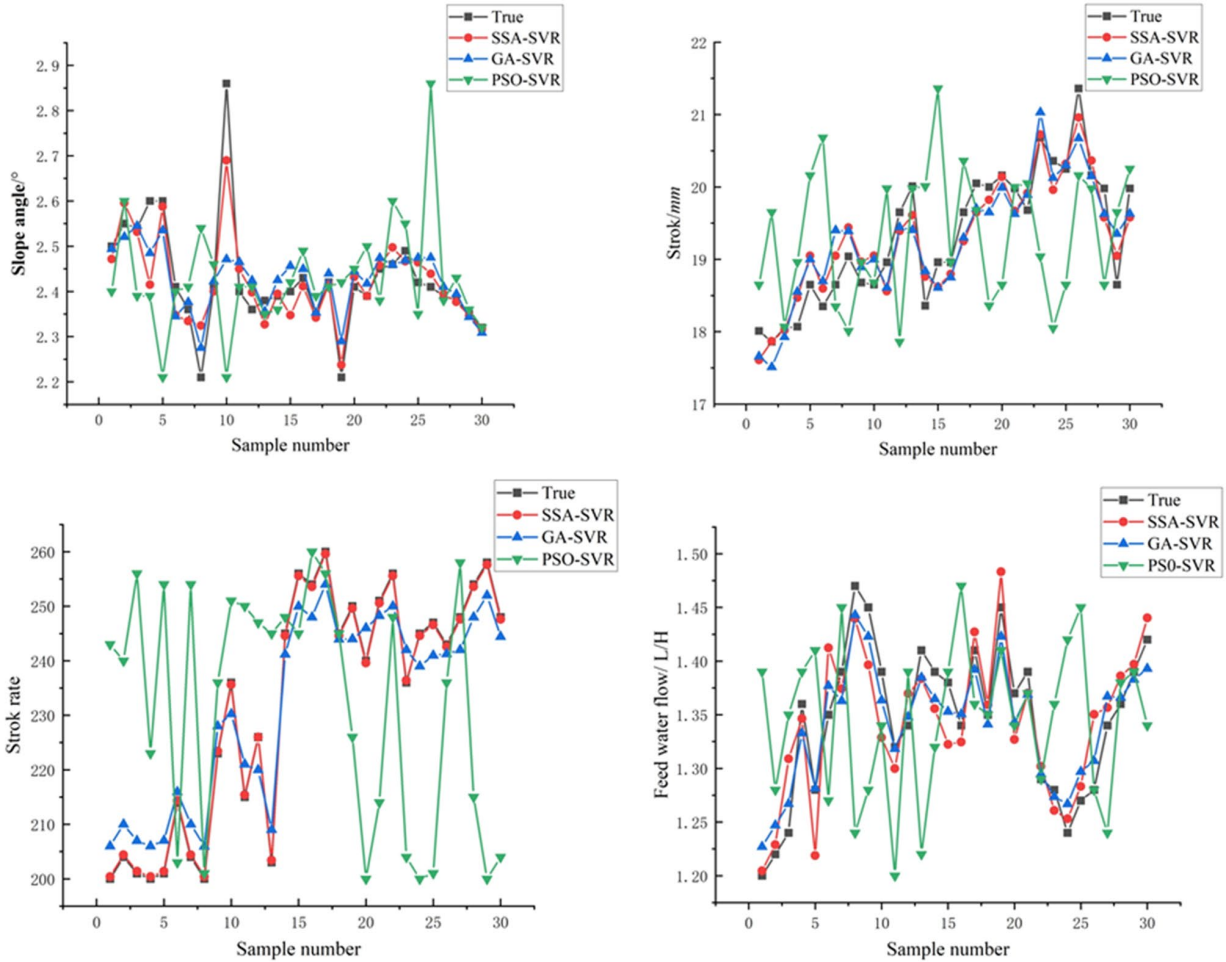
Simulation results of the models on control parameters of shaking table.

The five multi-scale eigenvalue groups of the ore belt images of the shaking table are used as the input of the model to predict the control parameters (such as stroke, stroke rate, feed water flow, and lateral slope angle) of the beneficiation shaking table and the work indicators of the beneficiation efficiency during the current operation of the entire equipment. As shown in Fig. 7, from the simulation results of the SSA-SVR, GA-SVR, PSO-SVR model on the control parameters of the shaking table, it can be seen that the SSA-SVR model has the best simulation effect on the control parameters, but the PSO-SVR model is the worst performer. Overall the GA-SVR model also performs well, with a good approximation to the true value.

**Figure 8:**
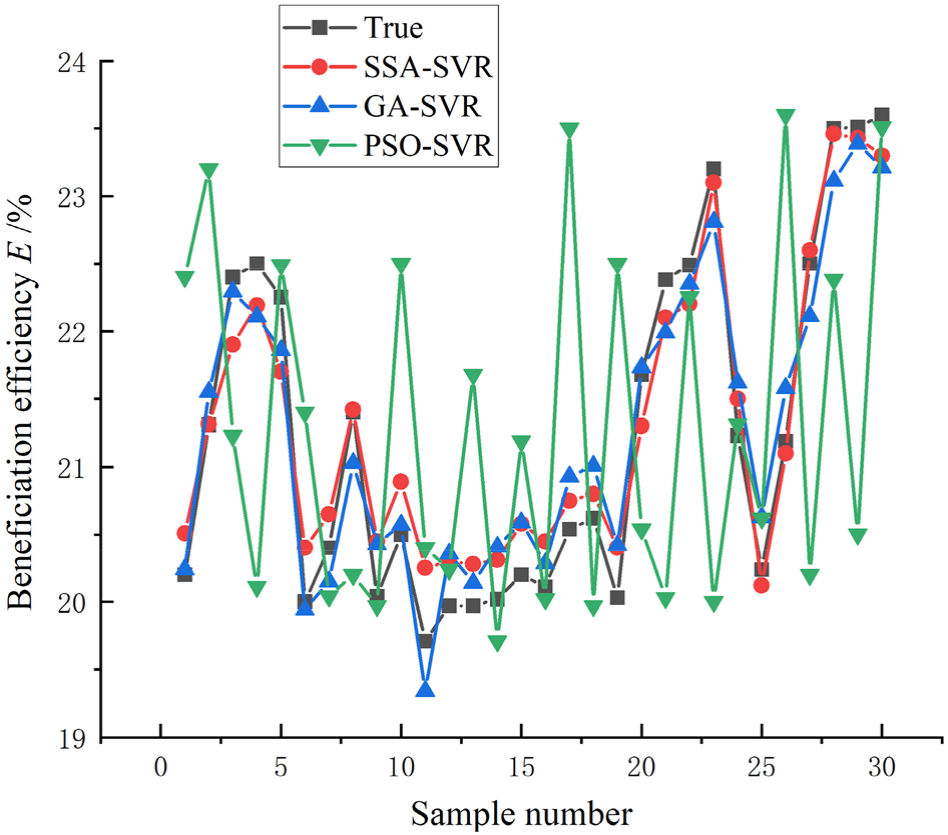
Simulation results of the models on beneficiation efficiency *E* of shaking table.

As shown in Fig. 8, from the simulation results of the beneficiation efficiency, the PSO-SVR model still performs poorly, with poorer learning ability compared to GA-SVR and SSA-SVR. A closer comparison shows that the SSA-SVR model performs slightly better than the GA-SVR model. As shown in Table 2, from the analysis of these index values of machine learning regression, the *MSE*, *RMSE*, and *MAE* values of the model predicted stroke, stroke times are all greater than the lateral flushing water and lateral slope, so the model prediction deviation of stroke times and strokes should be greater than the lateral fees water flow and lateral slope angle, and the prediction deviation of the model for beneficiation efficiency is at the average level. According to the evaluation index of *MAPE*, the prediction error of the SSA-SVR model for the stroke rate in the control parameters is the smallest, and it still maintains the most superior performance, and the prediction errors of other control parameters and beneficiation efficiency are basically the same. From the analysis of *R*^*2*^, the authenticity of the model prediction can be clearly seen. The SSA-SVR model has the highest accuracy in predicting stroke times, followed by beneficiation efficiency and stroke. However, on the whole, the *R*^*2*^ values are all greater than 0.85, indicating that the model has a relatively high level of accuracy and high prediction effects that meet precision requirements.

**Table 2:** Simulation result of the models.

SSA-SVR (GA-SVR)	*MSE*	*RMSE*	*MAE*	*MAPE*	*R*^*2*^
Feed water flow	0.00127 (0.0012)	0.03568 (0.042)	0.02950 (0.05422)	2.1899% (2.6532%)	0.86459 (0.86354)
Stroke	0.10196 (0.1895)	0.31931 (0.4563)	0.29074 (0.39540)	1.4769% (1.5987%)	0.91762 (0.92354)
Stroke rate	0.15194 (0.2154)	0.3898 (0.4356)	0.38018 (0.54231)	0.15614% (0.16521%)	0.99996 (0.98652)
Slope angle	0.00333 (0.0852)	0.0577 (0.1023)	0.036497 (0.09865)	1.471% (1.6521%)	0.88343 (0.87365)
Beneficiation efficiency	0.11575 (0.2153)	0.34022 (0.4120)	0.31696 (0.39856)	1.7902% (2.0132%)	0.93654 (0.91565)

**Figure 9:**
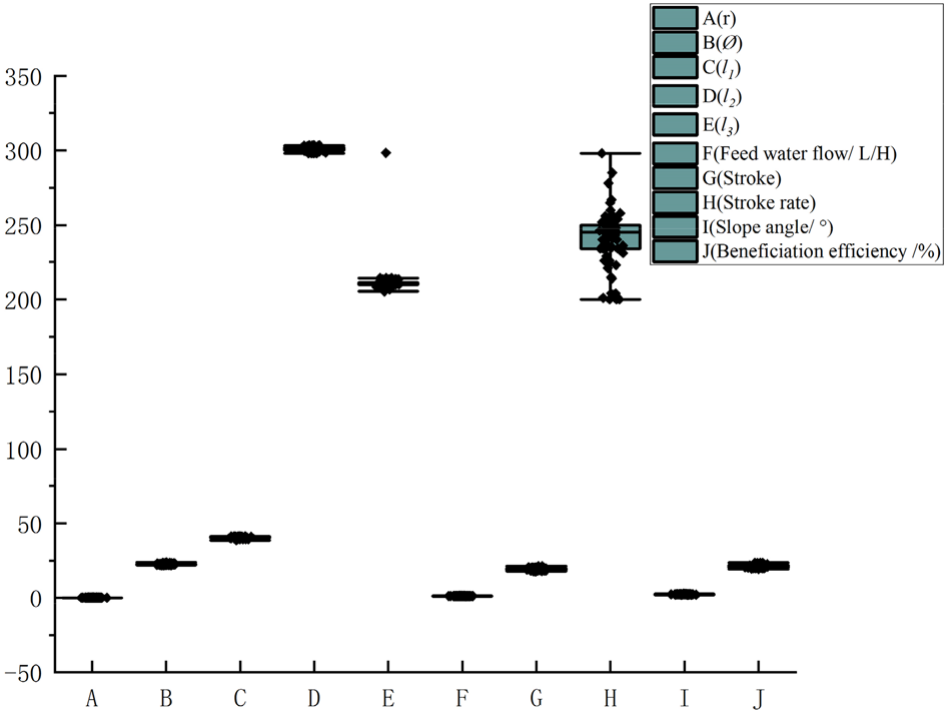
Abnormal analysis of control parameter values and ore belt characteristic.

Due to the small sample data of 80 pieces, the number ratio of test set and training set is not always appropriate, which may cause the problem of insufficient training depth of the model and affect the final practical application of beneficiation efficiency results. For this problem, the data sample was expanded and the model retrained to further validate the generalization and learning ability of the model and to avoid some errors due to the small sample size. As a result of the hard work of our staff and the support of the relevant technical staff, 80 sets of data were obtained again, so that, together with the previous 80 sets, the data from a total of 160 trials were available for this study. As shown in Fig. 9, the limited nature of the data obtained led to a high proportion of random data and some errors in the experimental process, resulting in a not very reasonable data analysis. By expanding the data, it was found that the distribution would be more reasonable when there was enough data.

In order to ensure that the training after data expansion is comparable to the training before expansion, the same ratio of training and test sets should be required. Therefore, 100 sets of data were used to train the learning process of the model and 60 sets of data were used to test the training results of the model. Given the small gap in simulation results between the GA-SVR model and the SSA-SVR model before the experimental data was extended, this may be due to the GA-SVR model being more data-dependent, resulting in the GA-SVR model not being trained deep enough. To ensure the scientific validity of our comparison, we retrained the GA-SVR model and the SSA-SVR model after the data had been expanded and again compared their simulation results in terms of control parameters and beneficiation efficiency of shaking table.

**Figure 10:**
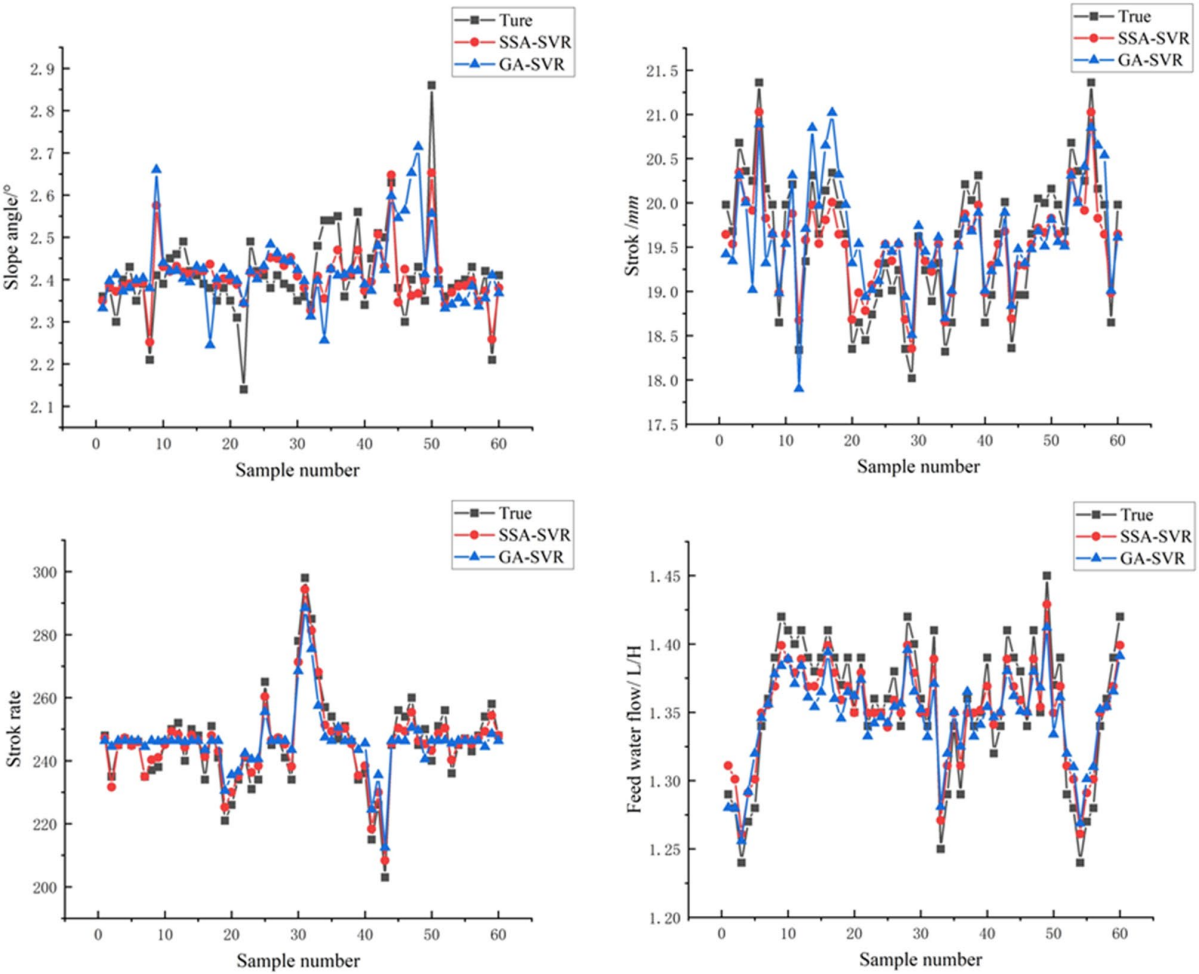
Simulation results of the models on control parameters of shaking table (To supplement the sample data and re-do the training for further verification).

**Figure 11:**
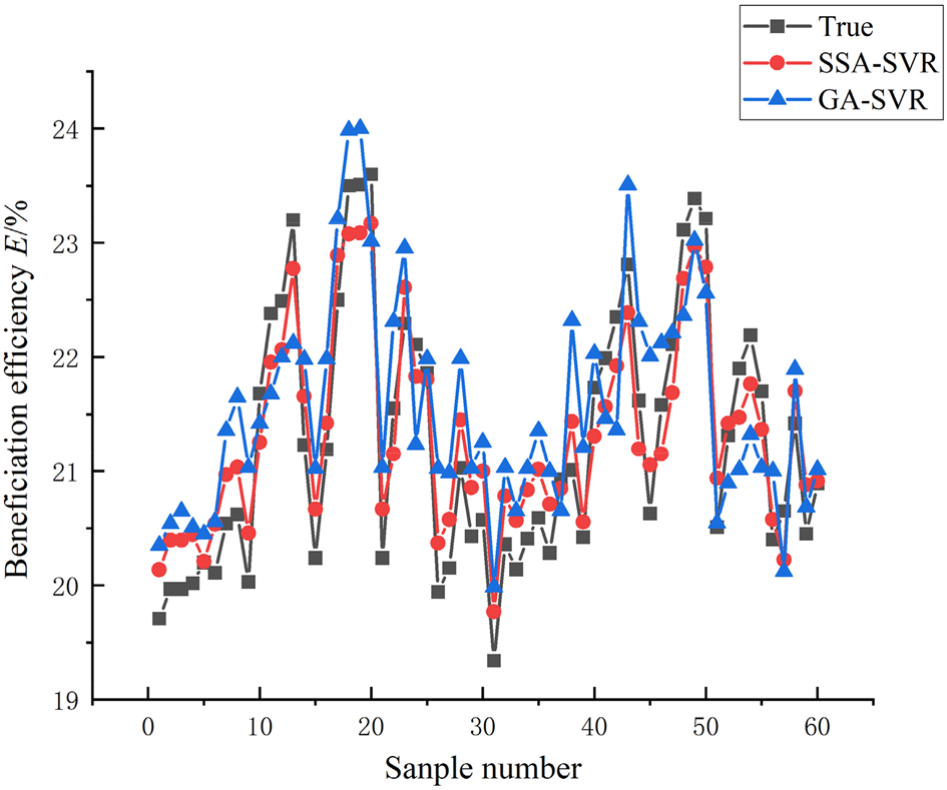
Simulation results of the models on beneficiation efficiency *E* of shaking table (To supplement the sample data and re-do the training for further verification).

As shown in Figs. 10 and 11, the simulation results of the GA-SVR model and SSA-SVR model for the operational parameters show that they still perform strongly, comparing to training with 50 sets of data, there are a little improvement in the closeness to the true value, which also shows that the GA-SVR model and SSA-SVR model learned from training with 100 sets of data are not greatly improved compared to training with 50 sets of data. As shown in Table 3, even with sufficient data, the SSA-SVR model still predicts better than the GA-SVR model, which is sufficient to demonstrate the superior ability of the SSA-SVR model in learning nonlinear relationships between geometry features of ore belt and control parameters, beneficiation efficiency.

**Table 3:** Simulation result of the models (To supplement the sample data and re-do the training for further verification).

SSA-SVR (GA-SVR)	*MSE*	*RMSE*	*MAE*	*MAPE*	*R*^*2*^
Feed water flow	0.0010 (0.0021)	0.03452 (0.0356)	0.028251 (0.02651)	2.1899% (2.21422%)	0.88957 (0.8754)
Stroke	0.10102 (0.2153)	0.32452 (0.4123)	0.28635 (0.28534)	1.4769% (1.51235%)	0.92356 (0.92215)
Stroke rate	0.15231 (0.1654)	0.37856 (0.3894)	0.39368 (0.38956)	0.15614% (0.16354%)	0.99896 (0.99452)
Slope angle	0.00325 (0.0045)	0.0545 (0.0895)	0.04235 (0.05324)	1.471% (1.56356%)	0.89865 (0.88632)
Beneficiation efficiency	0.11512 (0.1564)	0.3532 (0.3546)	0.37895 (0.38654)	1.7902% (1.8956%)	0.98214 (0.98201)

## Adaptive optimization method of shaking table control parameters based on maximization of beneficiation efficiency

It is known that through the advanced technologies of machine vision and machine learning, the multiscale geometric features of the ore belt of shaking table can be characterized as the separation indicators and control parameters with the highest degree of authenticity, so that the separation state and control parameters of the equipment can be monitored real-time through the ore belt. However, to truly realize the automatic optimization of the control parameters, these research results should be used to design a control algorithm or optimization method.

**Figure 12:**
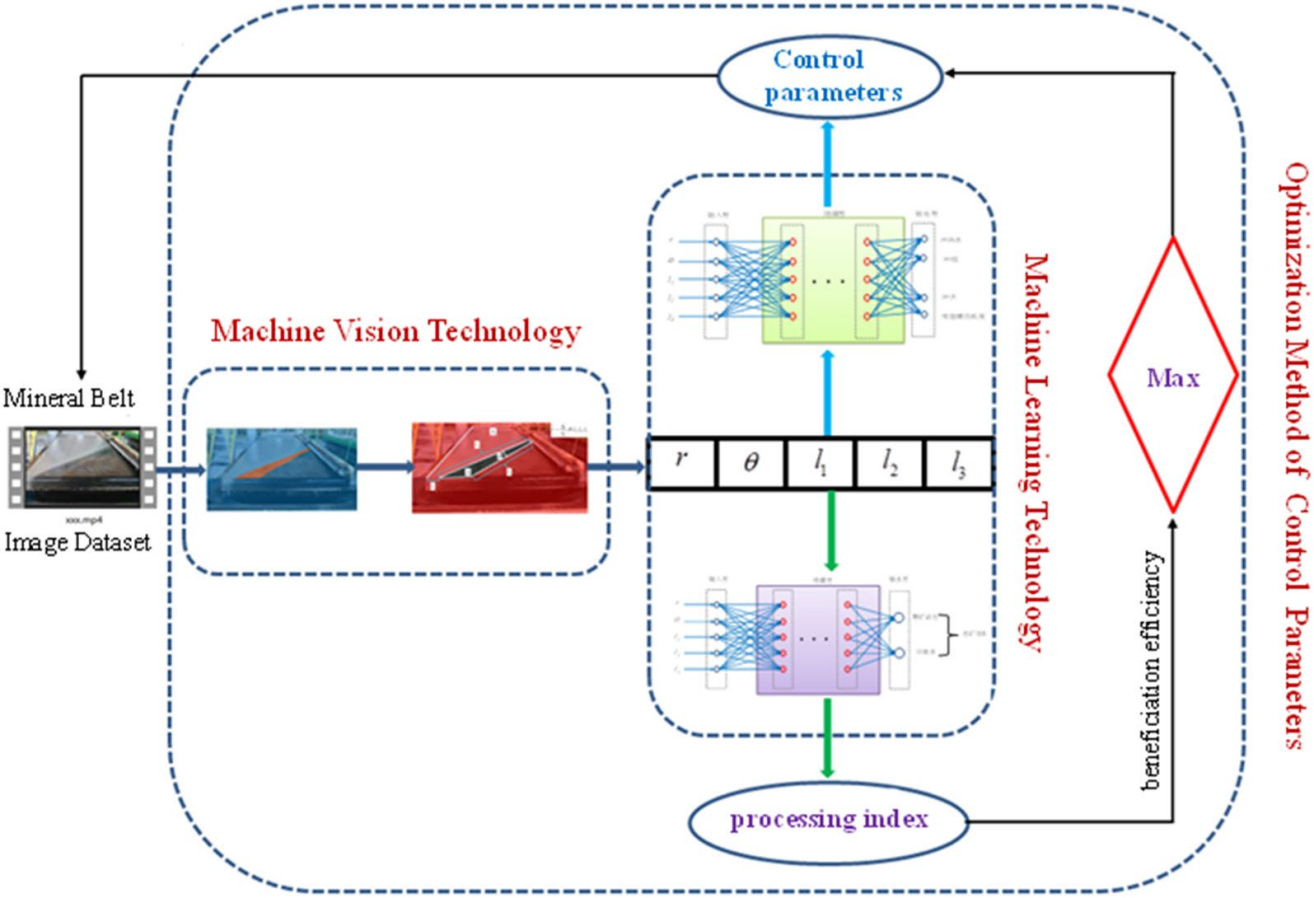
Research on optimization of shaking table control parameters combining machine vision and machine learning technology.

Therefore, after preliminary conception, we have roughly designed an adaptive optimization idea for the control parameters of the shaking table combining machine vision and machine learning technology. As shown in Fig. 12, the optimization idea is mainly divided into three steps. The first is the machine vision part, which mainly adopts the deep learning algorithm, aiming at obtain the multi-dimensional geometric feature values of the points, lines and surfaces of the predicted ore belt image. The second part is the data modeling part of machine learning, which uses the relational model of "image characteristics of ore belt and beneficiation efficiency" and the optimal control model of "image characteristics of zoning and control parameters" to dynamically monitor the separation status and fluctuations of control parameters. The last part is the design part of the optimization method, which mainly combines the principle of the highest beneficiation efficiency to optimize the control parameters adaptively.

### Design of optimization methods

In this paper, the design of the optimization method is the key to realizing the optimization of multiple control parameters. This optimization method can only be realized by combining the largest beneficiation efficiency. As shown in Fig. 15, according to the previous research results, we put forward the idea of adaptive optimization of control parameters of shaking table based on the highest beneficiation efficiency.

**Figure 13:**
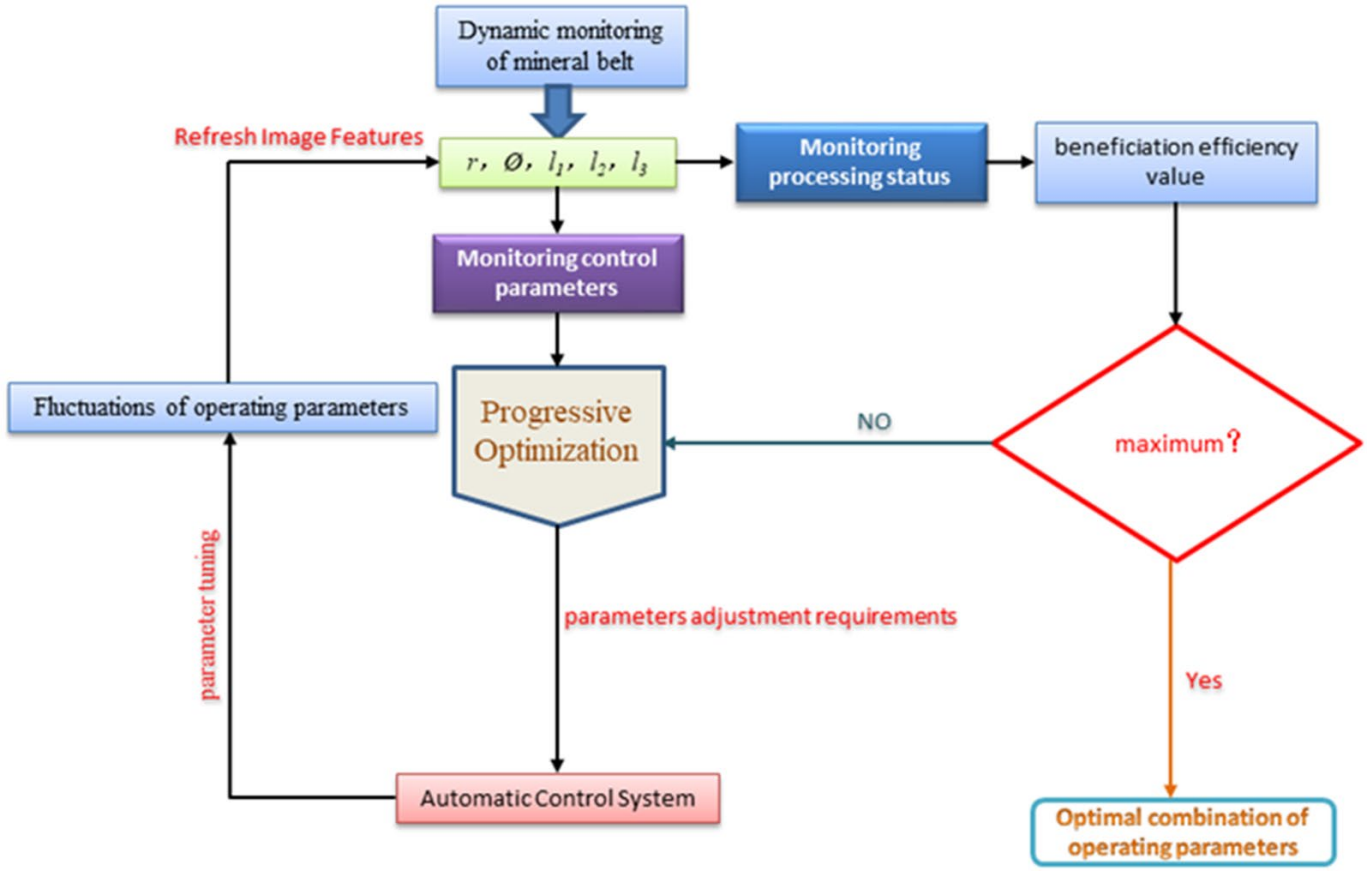
The self-adaptive optimization of control parameters of gravity separation shaking table based on maximization of beneficiation efficiency.

As shown in Fig. 13, a method for optimizing the combination of control parameters shaking table based on the highest beneficiation efficiency is designed. It can set reasonable indicators of beneficiation efficiency according to different requirements from plant. Through the image recognition system and data processing system to dynamically monitoring the beneficiation efficiency and fluctuations of equipment parameters, it is possible to judge in real time whether the current separation condition meets the requirements, and carry out the optimization procedure. If the current separation conditions meet the given index requirements of the concentrator, the current corresponding control parameter values of shaking table, such as feed water flow and lateral slope angle value, as well as the geometry eigenvalues of the ore belt, we can define these as the optimized equipment parameter values, which is the best combination of parameters for the beneficiation shaking table of this concentrator when separating this kind of minerals.

**Figure 14:**
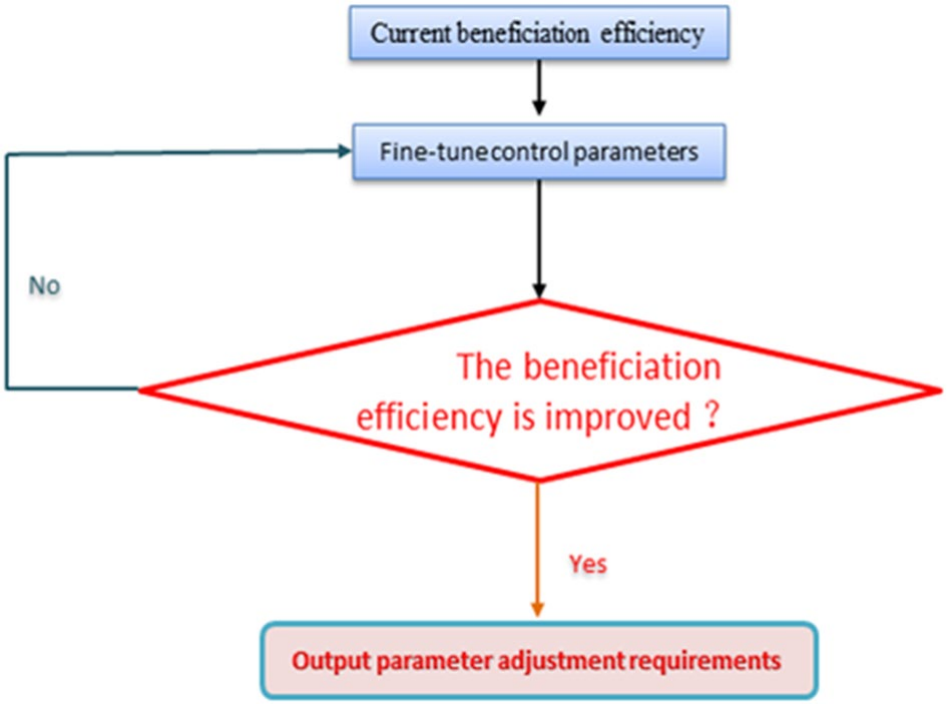
The principle of progressive optimization.

If the current separation conditions do not meet the requirements of the given index, it is necessary to carry out progressive optimization to provide the control system with instruction of parameter adjustment. However, after the control system fine-tunes the parameters, the image features of the ore belt are refreshed, thus completing the one optimization of beneficiation efficiency, and then the current beneficiation index is re-compared with the set index until each beneficiation status basically reaches the given standard. As shown in Fig. 14, the progressive optimization method has two obvious characteristics. first, it is a fine-tuning continuous optimization, and second, it aims to enhance the beneficiation efficiency. And it takes the current beneficiation efficiency as input and the control parameter value as output, by judging the current beneficiation efficiency, the control parameters are continuously fine-tuned until the beneficiation efficiency can be improved, and the current equipment parameters are output, otherwise, the motor continues to be controlled to fine-tune the parameters. And the characteristics of the ore belt fluctuate constantly in this process. After the new zoning characteristics appear, the combination of the new separation condition and control parameters will also appear, so as to realize continuous automatic parameter adjustment until it meets the index requirements of industrial mineral processing efficiency.

### Analysis of optimization results

**Figure 15:**
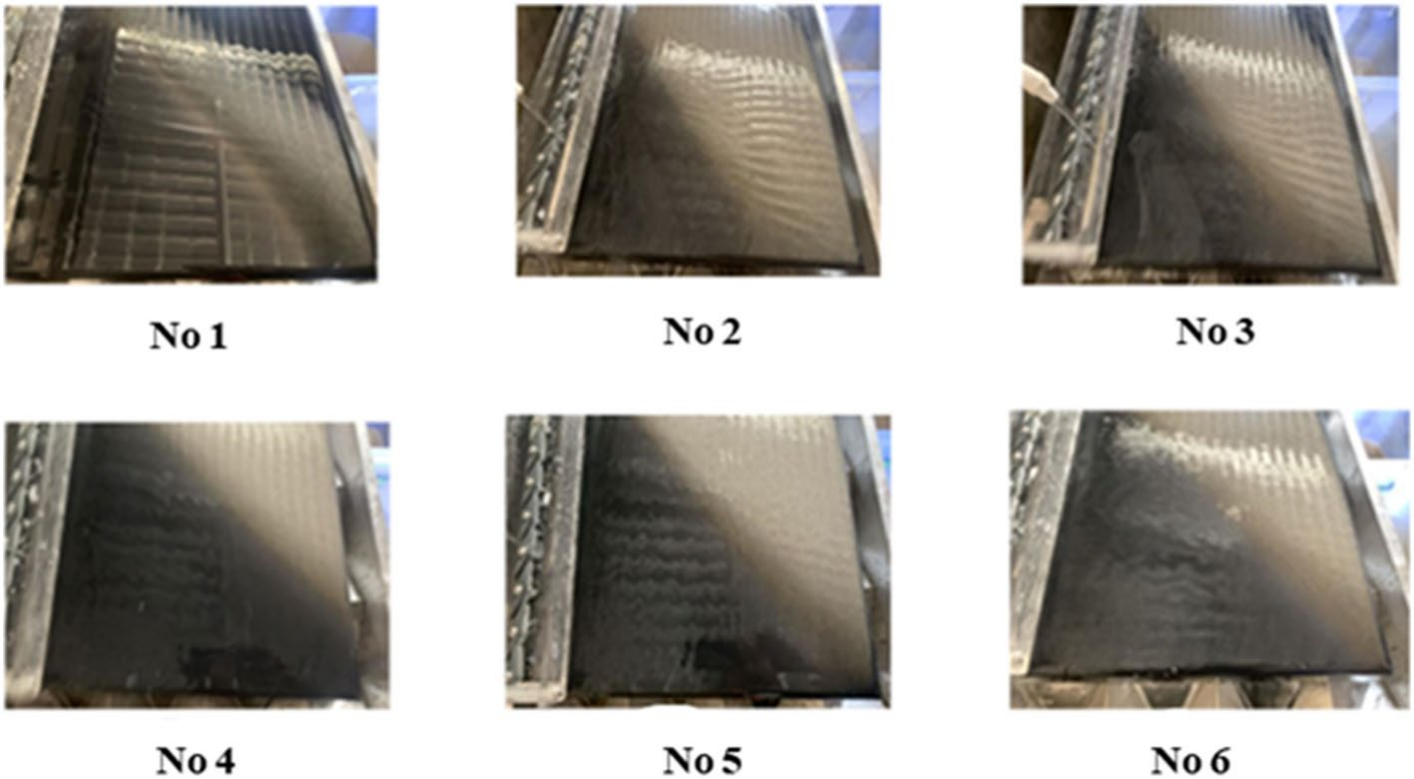
Six representative ore belt images to be optimized.

To verify the rationality and reliability of the self-adaptive optimization method of the control parameters of the beneficiation shaking table designed in this paper, we conducted six sets of experiments from the industrial shaking table sorting process. As shown in Fig. 15, to obtain six representative sets of experimental data to be optimized, we use six different shapes of ore belt distribution images as representatives to characterize the equipment operation and processing indicators under six extreme separation conditions. As shown in Fig. 15, due to the large range of parameter adjustment in the six groups of experiments, the fluctuation of the ore belt is also large, which just shows that the images of these ore belts are representative. We first numbered the 6 ore belt images in order, as shown in Table 4, we obtained the corresponding control parameter values and beneficiation efficiency values of each number, as shown in Table 5, the geometric eigenvalues of each ore belt image are automatically calculated by deep learning image processing technology, it can be seen that the characteristics of the ore belt fluctuate greatly regardless of which dimension of point, line, and surface, and the separating state also changes greatly.

**Table 4:** Control parameters to be optimized and current beneficiation efficiency E.

Sample number	Feed water/*ml/s*	Stroke/*mm*	Stroke rate/*r/min*	Slope angle/°	*E*(%)
1	44.9	18.01	200	3.0	31.78
2	43.7	17.86	204	1.0	42.78
3	65.1	18.05	201	2.1	30.23
4	30.7	18.05	201	3.5	71.65
5	30.4	18.05	201	2.5	34.23
6	59.9	18.05	201	2.5	57.33

**Table 5:** Current image features of ore belt to be optimized (the most representative 3D features are chosen in ore belt image).

Sample number	*R*	Ø/°	*l*_1_
1	0.059	23.7542	373
2	0.115	32.5689	197
3	0.150	38.5625	189
4	0.058	22.6547	455
5	0.079	23.5234	431
6	0.065	23.3564	470

As shown in Table 4, according to the actual situation of the concentrator, we only experimentally adjusted the lateral flushing water and lateral slope of the shaking table, and other control parameters were almost unchanged. The adjustment of the two control parameters is the most frequent and has the greatest impact on the beneficiation efficiency, so the urgency of optimization is also the strongest. Since the excessive control parameters are optimized, the cost of optimization will increase and the efficiency will decrease. Therefore, in order to apply our research results to industrial production practice as soon as possible, we will only carry out adaptive optimization of the control parameters for the feed water and slope angle of the shaking table, and at the same time, the image features of the currently optimized ore belt are also obtained.

These test data are imported into the designed optimization method and then observe the change of separating state before and after optimization, record the change of control parameters and the ore belt of the shaking table respectively, and calculate the data fluctuation before and after optimization. In actual production practice, it can be assumed that that a set requirements of separation state of a given system with a beneficiation efficiency of 72% are based on the beneficiation plant. It is found that there is no set of data in the input separation state that meets the requirements. Even though the fourth group is relatively close to the target beneficiation efficiency, the other groups of data are far from the beneficiation efficiency of the given demand. Therefore a certain number of successive optimizations are required to achieve the established targets. The experimental data before optimization is imported into the optimization system. After the system receives the current separation conditions of beneficiation efficiency value, it finds that the requirements are not met, so the progressive optimization provides the parameter adjustment requirements, after the control parameters are adjusted by the control system, the geometry shape of the ore belt also changes. And the image processing software extracts rich zoning features, the model simulation system predicts the specific values of the control parameters and the separation state, then we record the system predictions data value of various indicators in turn.

**Figure 16:**
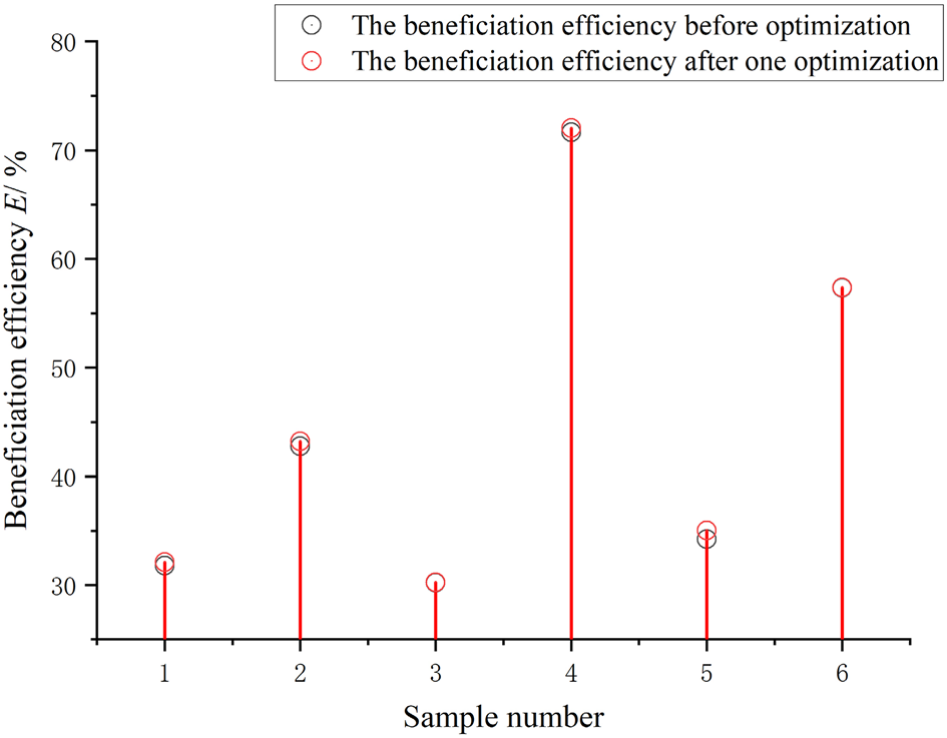
The fluctuation of beneficiation efficiency after one optimization.

**Figure 17:**
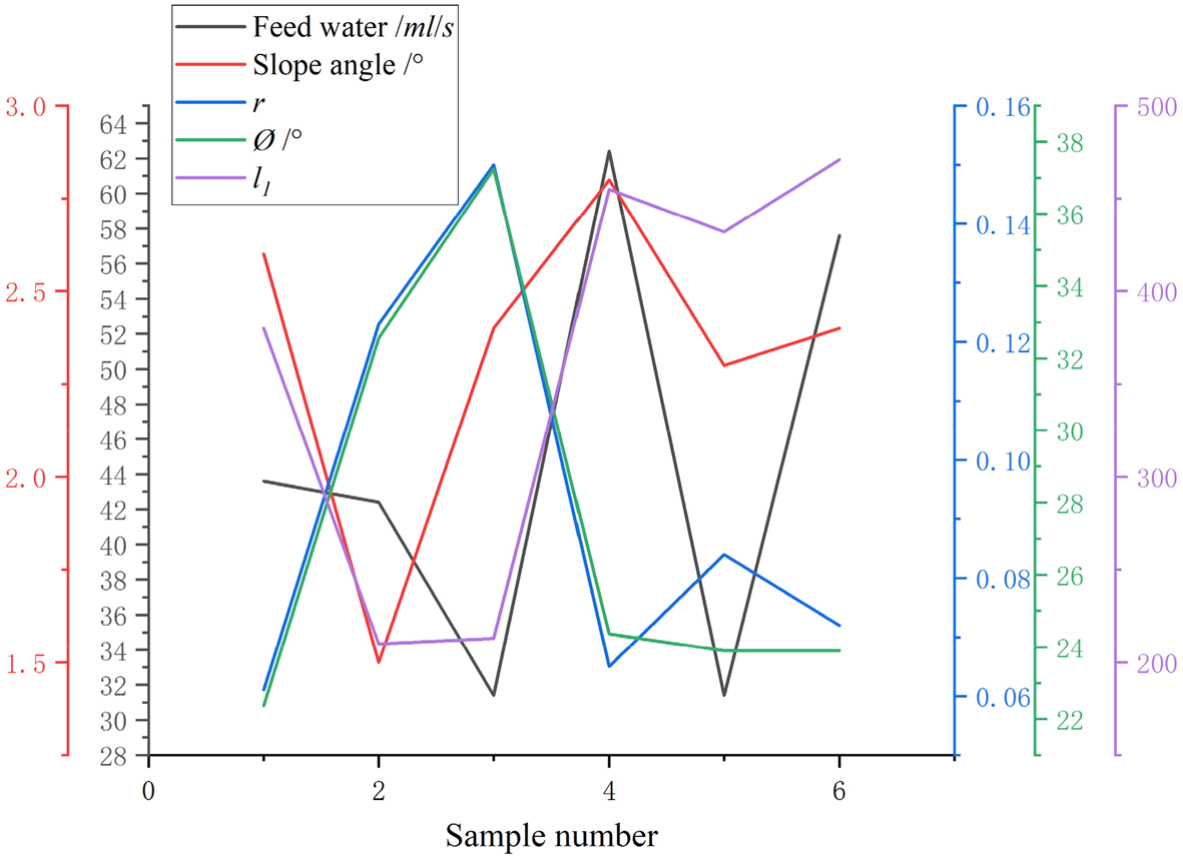
The fluctuation of characteristic parameter value of ore belt of shaking table after one optimization.

As shown in Fig. 16, by comparing the beneficiation efficiency before and after optimization, it is found that although the fluctuation is not large, the beneficiation efficiency of each set of test data after optimization is slightly higher than the before optimization, which fully proves that the optimization process is continuous and gradual. Although the optimization efficiency is slow, the optimization results are guaranteed. The fluctuations of the control parameter values of the shaking table and the characteristic values of the ore belt after one optimization are shown in Fig. 17. It can be seen that the fluctuation trend of the two control parameter values and the three characteristic values of ore belts after the one optimization is relatively consistent, which proves that the optimization method proposed in this paper does not change the attribute relationship between the parameters of the shaking table, and the optimization process is very stable. Since the beneficiation efficiency has been improved to meet the given index requirements after one optimization, we only record the data values after one optimization. As shown in Table 6, among the six groups of beneficiation efficiency data values after one optimization, the fourth group of beneficiation efficiency has met the requirement of 72% beneficiation efficiency. Therefore, the fourth group of optimized control parameter values and characteristic parameter value of ore belt are currently optimized operating parameters of the shaking table.

**Table 6:** Combination of control parameters and characteristic parameters of ore belt after one optimization.

Sample number	Feed water/*ml/s*	Slope angle/°	*r*	Ø/°	*l*_*1*_	*E*/%	Improvements/%
1	43.6	2.6	0.061	22.3658	380	**32.12**	1.070
2	42.4	1.5	0.123	32.5624	210	**43.21**	1.005
3	31.4	2.4	0.150	37.2547	213	**30.25**	0.660
**4**	**62.4**	**2.8**	**0.065**	**24.3654**	**455**	**72.03**	0.530
5	31.4	2.3	0.084	23.895	432	**35.02**	2.310
6	57.6	2.4	0.072	23.8932	471	**57.36**	0.052

**Figure 18:**
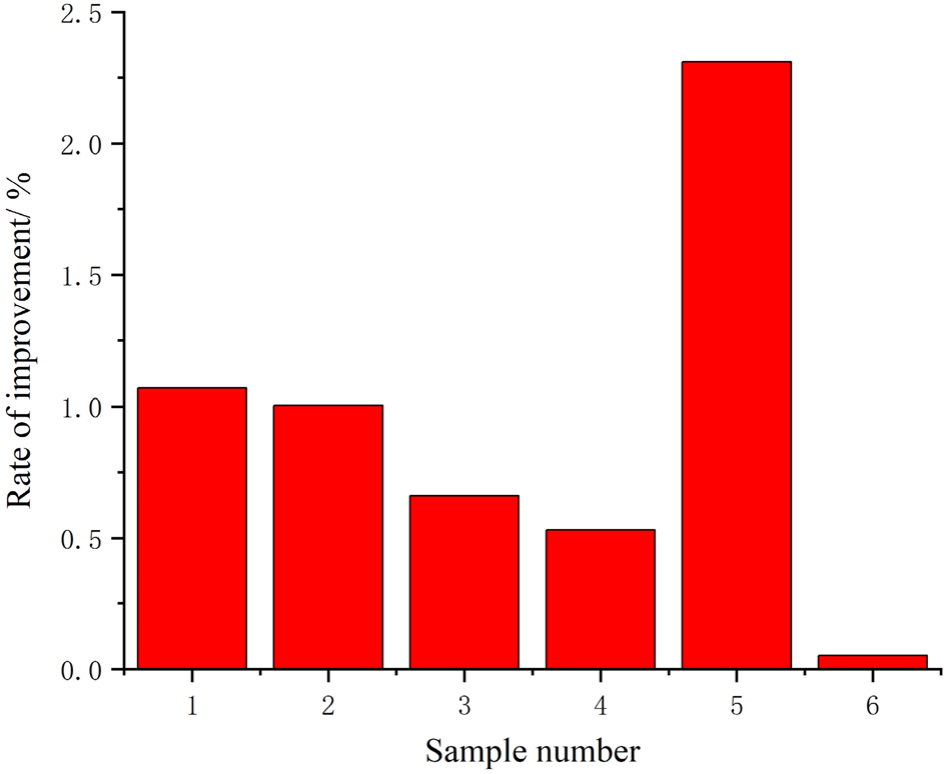
Rate of improvement in beneficiation efficiency after one optimization.

As shown in Fig. 16, by comparing the separating states before and after optimization, it was found that there was little fluctuation, but the post-optimization beneficiation efficiency was slightly higher than the pre-optimization efficiency. Although the variation is small, it shows that the optimization is a continuous and progressive process and thus the optimization results are guaranteed. As shown in Fig. 17, after the beneficiation efficiency has been optimised, the geometry of the corresponding ore zone is characterized by a combination of the optimized characteristic parameters. As shown in Fig. 18, although the fourth group has the lowest rate of improvement in beneficiation efficiency, it still meets the set requirements after one optimization, which also indicates that the later optimization rates are limited if the current beneficiation efficiency is high.

## Conclusions

At present, most of the adjustment of the control parameters of the gravity shaking table depends on workers’ observation and analysis of ore belt images, and then complete the judgment based on experience. As the knowledge level, technical experience and work responsibility of workers will inevitably be inconsistent, manual operation will also make the beneficiation efficiency more uncertain, which will lead to the increase of labor intensity to a certain extent, as well as the problems of unsatisfactory work effect and low work efficiency. The most critical problem is that it is difficult to ensure the best separation state of the shaking table.

Although the current research results only achieve the goal of reducing the burden on workers and preventing the loss of concentrate grade, and can only realize the research on automatic interception of concentrates, which is far from the real intelligent beneficiation shaker. Some studies have been adopted advanced deep learning image processing technology and machine learning data modeling method, but there is a lack of advanced research on the integration of the two, and the problems of optimal adjustment of control parameters and uncertainty of separation state still exist. In response to these problems, this paper has done the following work:

1. Through the deep learning segmentation algorithm, the multi-scale features of ore belt on the shaking table are extracted. In an experimental system incorporating this vision technique, we obtained a reasonable and considerable data sample of feature data from ore belt.
2. Combined with the research results of the first step, through the data-drive SSA-SVR method, the rela-tionship model between the characteristics of the ore belt and the control parameters of shaking table, as well as the control model between the characteristics of the ore belt and the separating state are obtained, and the real mapping of the ore belt image to the control parameters and the beneficiation efficiency is acquired, which resolved the nonlinear relationship between the internal and external property parameters of the shaking table.
3. The most important thing is that this paper integrates the research results of the previous two steps, and proposes an adaptive optimization method of control parameters based on the maximization of beneficiation efficiency, which realizes the continuous optimization of the separating state, and finally obtained the current optimized parameter combination of control parameters and characteristics of ore belt of shaking table.
4. The research results of this paper have laid the foundation for the intelligent control of the production of shaking table and the development of the intelligent shaking table. If the research results are integrated with the automatic control systems, such as the self-adaptive interception system of the mineral processing products of the shaking table, and the automatic adjustment of the slope angle, and the flushing water, it will be able to develop an intelligent mineral processing of shaking table with controllable quality of mineral processing products and the highest beneficiation efficiency, which is also the next research goal of the team.

## Supplementary Information

Supplementary Information 1.

## Data Availability

The datasets used and analyzed during the current study available from the corresponding author on reasonable request.The datasets generated and analyzed during the current study are not publicly available due to the project interests and the original technology protection of schools and enterprises but are available from the corresponding author on reasonable request.
