# Supplementary figures and images for: Research on optimization of control parameters of gravity shaking table
Source: Sci Rep. 2023 Jan 20;13:1133. doi: 10.1038/s41598-023-28171-5 (PMC9860046; doi:10.1038/s41598-023-28171-5)

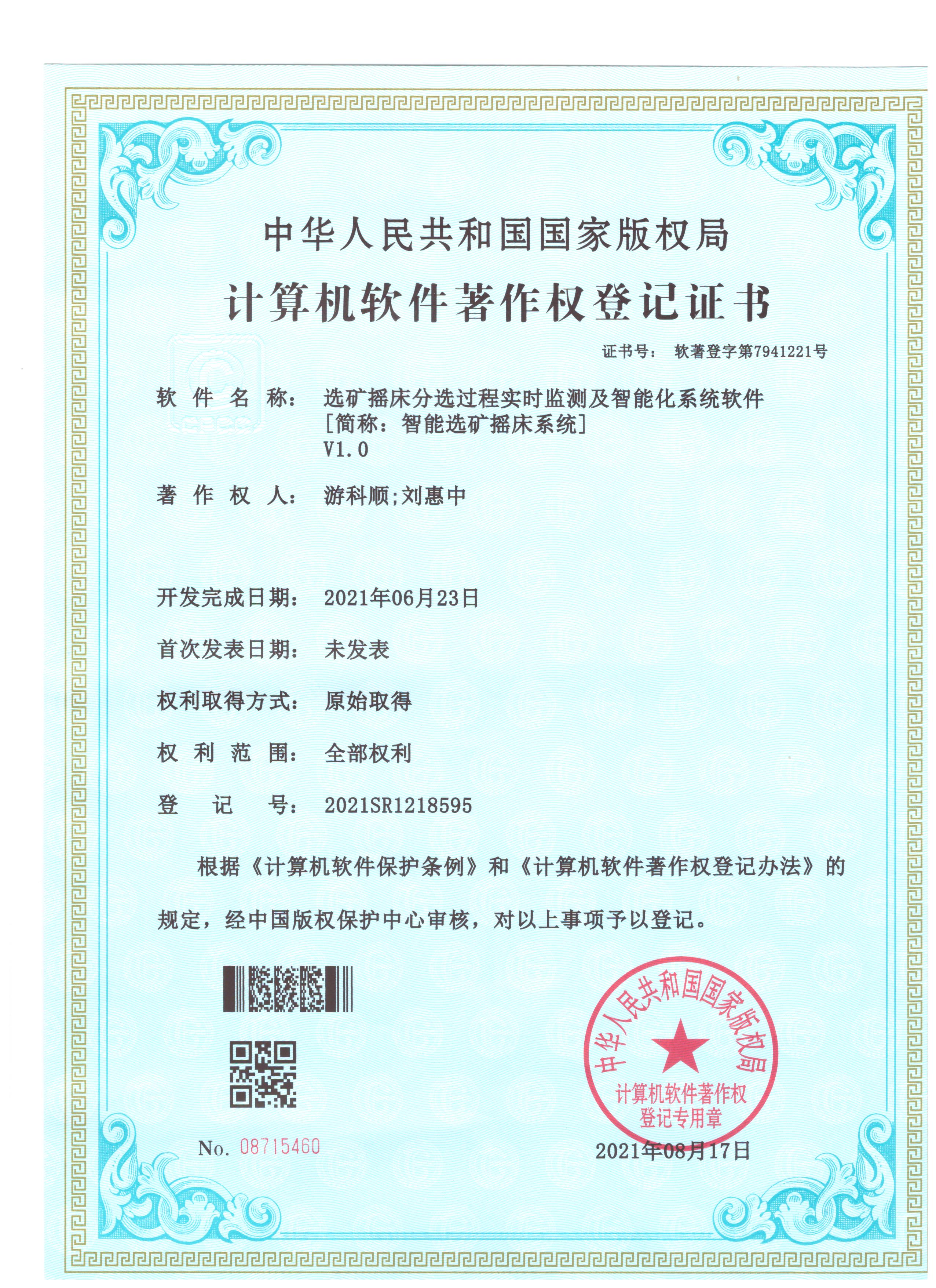

Supplement: Supplementary file 1 — Supplementary Information 1. [file 41598_2023_28171_MOESM1_ESM.jpg]
